# Identification and Characterization of Four Novel Viruses in *Balclutha incisa*

**DOI:** 10.3390/insects15100772

**Published:** 2024-10-06

**Authors:** Jiajing Xiao, Guang Yang, Renyi Liu, Danfeng Ge

**Affiliations:** 1 Center for Agroforestry Mega Data Science, Haixia Institute of Science and Technology, Fujian Agriculture and Forestry University, Fuzhou 350002, China; jjxiao@fafu.edu.cn (J.X.); 1220514077@fafu.edu.cn (G.Y.); 2 College of Life Science, Fujian Agriculture and Forestry University, Fuzhou 350002, China

**Keywords:** *Balclutha incisa*, novel viruses, ollusvirus, iflavirus, totivirus

## Abstract

**Simple Summary:**

*Balclutha incisa* is a widespread leafhopper found in the fields of rice and other crops, whose feeding habits and potential for pathogen transmission pose a threat to crop production. However, there is limited information about the viruses that *B. incisa* may carry. In this study, we used deep sequencing technology to examine the virome of *B. incisa* and reported the complete genomes of four novel viruses using rapid amplification of cDNA ends (RACE). Among these, B. incisa ollusvirus 1 (linear) and B. incisa ollusvirus 2 (circular) both exhibit the typical G-N-L genome structure characteristic of the order *Jingchuvirales*. Additionally, we identified two other viruses, B. incisa iflavirus 1 and B. incisa totivirus 1. Phylogenetic and sequence identity analyses suggest that these viruses represent new members of their respective families. Our study provides valuable insights into the virome of *B. incisa* and identifies four new viruses that may be potentially harmful to crops.

**Abstract:**

*Balclutha incisa* (Cicadellidae: Deltocephalinae), a leafhopper prevalent in tropical and temperate regions, is notably abundant in grasses and rice. The virome of *B. incisa* was investigated using deep transcriptome sequencing, leading to the first identification of four viruses belonging to the families *Aliusviridae*, *Iflaviridae*, and *Totiviridae* in *B. incisa*. These viruses have been provisionally named B. incisa ollusvirus 1 (BiOV1), B. incisa ollusvirus 2 (BiOV2), B. incisa iflavirus 1 (BiIV1), and B. incisa totivirus 1 (BiTV1). The complete genome sequences of these viruses were obtained through rapid amplification of cDNA ends (RACE). BiOV1 has a linear genome of 15,125 nucleotides (nt), while BiOV2 possesses a circular genome of 14,853 nt. The BiIV1 genome, excluding the poly(A) tail, is 10,903 nt in length and encodes a single open reading frame (ORF) for a polyprotein consisting of 3194 amino acids (aa). The BiTV1 genome is 4357 nt long and contains two overlapping ORFs, with the viral RNA-dependent RNA polymerase (RdRp) translated via a −1 ribosomal frameshift. Phylogenetic and sequence identity analyses suggest that all these viruses are novel members of their respective families. This study significantly expands our understanding of the virome associated with *B. incisa* by reporting and characterizing these novel viruses.

## 1. Introduction

Rice (*Oryza sativa* L.) is a cornerstone of global food security, serving as a staple food for a significant portion of the world’s population [1,2]. Despite its critical role in global food security, rice cultivation faces numerous challenges, among which are the threats from pests and related viral diseases [3,4]. Among the most damaging viral diseases are those transmitted by insect vectors, particularly leafhoppers, planthoppers, and treehoppers, which play critical roles in spreading these pathogens [5,6]. For instance, Rice stripe virus (RSV) and Rice black-streaked dwarf virus (RBSDV), both prevalent in Asia, are primarily transmitted by the small brown planthopper (*Laodelphax striatellus*) [7,8,9]. Rice dwarf virus (RDV) is transmitted by the green rice leafhopper (*Nephotettix cincticeps*), which transmits the virus in a persistent, circulative-propagative manner, ensuring long-term infection [10,11]. Southern rice black-streaked dwarf virus (SRBSDV), also found in East and Southeast Asia, is mainly spread by the white-backed planthopper (*Sogatella furcifera*), which is also known to transmit other viral groups like totiviruses and Solemo-like viruses [12,13,14]. Rice yellow mottle virus (RYMV), a major virus in Africa, is transmitted by various beetle species and grasshoppers rather than leafhoppers [15]. Rice stripe necrosis virus (RSNV), which affects rice in both Africa and America, is transmitted by the soil-borne fungus *Polymyxa graminis* [16]. Understanding the specific insect vectors of these viruses is essential for tracing the transmission dynamics of these viral diseases. This knowledge is critical for developing effective strategies to manage and prevent outbreaks in rice cultivation, thereby safeguarding global food security.

Leafhoppers are notorious vectors for a variety of viral pathogens that infect rice and other economically important crops, including wheat, tomatoes, and peppers [17]. *Balclutha incisa* is a widely distributed leafhopper frequently found on rice and grasses [18]. It closely resembles *Balclutha rubrostriata* in appearance, though the two species can be distinguished by the morphology of the aedeagus [19]. *B. incisa* has been identified as a vector for the ‘*Candidatus* Phytoplasma solani’ strain, associated with ‘bois noir’ in grapevine [20,21]. Despite this, the virome associated with *B. incisa* remains largely unexplored, and the potential viral threats it may transmit to crops are not well understood.

Traditional virus detection methods have been foundational in virology, serving as cornerstones in the quest to understand and combat viral infections [22,23]. Molecular techniques, metagenomics, and other high-throughput methods have emerged as powerful tools to overcome the limitations of traditional detection methods, enabling researchers to explore the vast and dynamic world of viruses more comprehensively [24,25]. Numerous viruses have been identified and characterized through sequencing reads from symptomatic and asymptomatic hosts, which greatly expands people’s awareness of the number and types of viruses [26,27,28,29]. Metagenomics circumvents this limitation by extracting and sequencing DNA directly from environmental samples, enabling the study of previously inaccessible microorganisms [30,31]. Beyond identifying species, metagenomics allows researchers to analyze the functional capabilities encoded in microbial genomes. This provides insights into the roles microorganisms play in biogeochemical cycles, nutrient cycling, and other ecological processes.

*Jingchuvirales* is a newly characterized order with non-segmented linear/circular or bisegmented linear/circular negative-sense RNA genomes, each harboring two to four open reading frames (ORFs) [32,33]. According to the International Committee on Taxonomy of Viruses (ICTV), *Aliusviridae* is a family of negative-sense RNA viruses in the range of 9.9–15.3 kilobases (kb) in length within the order *Jingchuvirales* and includes two genera, *Obscuruvirus* and *Ollusvirus*. Aliusviruses contain ORFs encoding glycoprotein (GP), nucleoprotein (NP), and a large protein with the RNA-dependent RNA polymerase (RdRp) domain (L), while some other members of this family also have a fourth ORF encoding a hypothetical protein. Viruses within this family are known to infect a range of arthropods, particularly insects, though the specific hosts and ecological impacts of many *Aliusviridae* viruses are still under investigation [34,35]. *Iflaviridae*, belonging to the order *Picornavirales*, contains a single genus iflavirus, which always contains a single-stranded RNA genome of 9–11 kb [36]. The genome includes a single ORF that encodes a large polyprotein of approximately 3000 amino acids (aa) in length. The polyprotein is post-translationally processed by the viral protease to produce mature polypeptides with functional conserved motifs [37]. The family *Totiviridae* consists of double-stranded RNA (dsRNA) viruses that primarily infect fungi and protozoa, though some members have been found in arthropods [38,39]. The genomes of these viruses often contain overlapping ORFs, encoding the capsid protein (CP) and RdRp. In the family *Totiviridae*, totivirus comprises a single linear molecule of the non-segmented genome, approximately 4.6 to 7.0 kb in length. At least three strategies for RdRp expression are found in *Totiviridae*: (1) as a fusion with CP via ribosomal frameshifting, (2) as a CP fusion without frameshifting followed by proteolytic release, and (3) as a separate protein via a termination-reinitiation mechanism [38].

Since leafhoppers are key vectors for virus transmission, the identification of novel viruses associated with rice leafhoppers provides information for early detection and better pest management strategies and helps safeguard agricultural productivity. In this study, *B. incisa* specimens were collected from rice fields in Yunxiao, Zhangzhou, Fujian Province, where rice viral diseases have been previously reported [6]. Using deep sequencing, we analyzed the virome of *B. incisa* samples and found 117 viral sequences, encompassing both RNA and DNA viruses. Among these sequences, we obtained the complete genome sequences of four viruses: B. insica ollusvirus 1 (BiOV1), a linear negative-sense single-strand RNA virus with a 15,125 nt genome; B. insica ollusvirus 2 (BiOV2), a circular negative-sense single-strand RNA virus with a 14,583 nt genome; B. insica iflavirus 1 (BiIV1), a linear positive-sense single-strand RNA virus with a 10,903 nt genome; and B. insica 1 (BiTV1), a linear double-strand RNA virus with a 4357 nt genome. These viruses represent novel members of their respective families, marking the first report of their presence in *B. incisa*. These findings provide valuable virome data that will be crucial for future research aimed at controlling potential pathogenic diseases transmitted by *B. incisa*. 

## 2. Materials and Methods

### 2.1. Sample Collection and Sequencing

The *B. incisa* samples were collected from a rice field in Yunxiao, Zhangzhou, Fujian Province, on 11 June 2021. After being caught in the field, the insect samples were temporarily reared on disease-free tender rice leaves. The collected insect samples were then photographed and screened under a microscope. The specimens were characterized by their uniform green body color and lacked the distinct reddish longitudinal stripes seen in related species such as *Balclutha rubrostriata* [19]. Adult samples were frozen in the −80 °C freezer. Four adults of *B. incisa* were randomly selected, and total RNA was extracted using TRIzol reagent (Invitrogen, Thermo Fisher Scientific, Waltham, MA, USA) according to the manufacturer’s instructions. The RNA-seq library was constructed with rRNA removed, and high-throughput sequencing of the samples was performed using the MGISEQ-2000 platform.

### 2.2. Genome Assembly and Virus Discovery

After obtaining the sequencing data, the 100-bp paired-end raw reads were filtered and trimmed using fastp (version 0.23.2) [40]. All contigs were de novo assembled using Trinity (version 2.1.1.0) with default parameters [41]. Subsequently, the virus sub-library of the National Center for Biotechnology Information (NCBI) non-redundant protein (nr) library was used for a BLASTx [42] search to screen candidate virus contigs (e-value < 10^−4^), and then the complete nr library was used to filter out non-viral biological sequences (e-value < 10^−4^).

### 2.3. Determination of Viral Genomes

To obtain the full-length sequence of viruses, we acquired the complete terminal sequences of virus genomes using 5′ and 3′ Rapid Amplification of cDNA End (RACE). In the 5′/3′ RACE processes, we used both the Random Primer and oligo (dT) primers from the Smarter RACE 5′/3′ Kit (Takara Bio, San Jose, CA, USA) to synthesize the first 5′/3′ RACE cDNA strand from the original RNA sample. Additionally, we synthesized the first 3′ RACE cDNA strand using the SMART RACE cDNA Amplification Kit (New England Biolabs, Ipswich, MA, USA), and the reverse transcriptase used was Protoscript II Reverse Transcriptase (New England Biolabs, Ipswich, MA, USA). Based on the two types of 3′ RACE cDNA, the virus terminal sequence was further amplified to confirm whether there was a poly(A) tail at the 3′ end.

We designed pairs of sequence-specific primers and carried out nested PCR. SuperTaq PCR StarMix (GenStar, San Francisco, CA, USA) containing high-purity Taq DNA Polymerase was used in the process. After obtaining amplicons of the target size, the amplicons were purified and cloned into the pMDTM 19-T vector (Takara Bio, USA), which were then transformed into *Escherichia coli* (*E. coli*). Following successful transformation, plasmid DNA was isolated from the transformed *E. coli* colonies and used for Sanger sequencing. We designed a pair of divergent primers at both ends of the BiOV1 and BiOV2 genomes for PCR to verify the possible circularity of the virus genomes, and the circularity of the genome was determined according to the sequencing results. The primers used are listed in Table S1.

### 2.4. Genome Annotation and Phylogenetic Analysis

ORFfinder (https://www.ncbi.nlm.nih.gov/orffinder (accessed on 20 November 2023)) was used to predict ORFs and their amino acid sequences in novel virus genomes. The predicted ORFs were preliminarily annotated using HHpred, combined with homology search results from BLASTp [43]. Conserved domains within the ORFs were predicted using CD-search from NCBI (https://www.ncbi.nlm.nih.gov/Structure/cdd/wrpsb.cgi (accessed on 20 November 2023)). Multiple sequence alignment was performed using MAFFT (version 7.464) [44] with default parameters, and sequence identities were calculated in R (version 4.2.3). IQ-TREE2 was used to construct phylogenetic trees based on the maximum likelihood method with the best-fit model and 1000 bootstraps [45]. Finally, the interactive Tree of Life (iTOL) was used to enhance the visual appearance of the phylogenetic trees [46]. For ollusviruses, amino acid sequences of the L protein from 40 viruses belonging to the order *Jingchuvirales* were obtained from NCBI, with the L protein of Sanya iflavirus 3 (GenBank No. MZ209879) used as the outgroup. In the phylogenetic analysis of the novel iflavirus, amino acid sequences from 43 viruses belonging to the family *Iflaviridae* were downloaded from NCBI, with poliovirus (GenBank No. NC_002058) used as the outgroup. For the phylogenetic analysis of totivirus, the amino acid sequences of both RdRp and CP from 37 viruses belonging to the family *Totiviridae* were obtained from NCBI. The corresponding amino acid sequence of Giardia lamblia virus (GenBank No. NC_003555) was used as the outgroup.

## 3. Results

### 3.1. Virome Analysis of B. incisa

**Figure 1:**
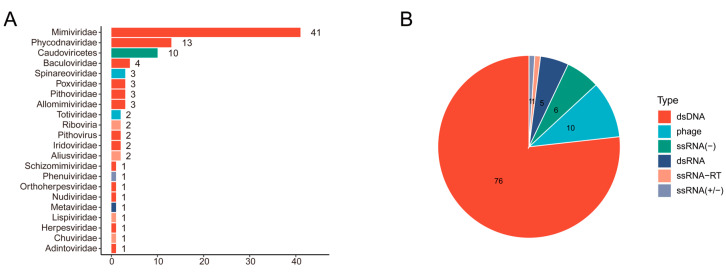
Taxonomic distribution of viruses detected from *B. incisa*. (**A**) Each horizontal bar represents the number of viruses in each family detected in *B. incisa*. (**B**) The number of different types of viruses belonging to dsDNA, ssRNA (−), ssRNA (+), ssRNA (−/+), phage, and unknown virus.

Since *B. incisa* is a common insect found in rice and other plants, we collected adult insects from the rice field and performed deep transcriptome sequencing to investigate the virome of this insect. A total of 78.32 million 100-bp paired-end reads were used for the assembly of 333,878 contigs. Viral-like contigs were screened using a BLASTx search against the NCBI Viral RefSeq database, and contigs with hits shorter than 500 nt or with the best hits from non-viral genomes were removed. Only 117 contigs were selected as candidate viral sequences, including 41 from *Mimiviridae*, 13 from *Phycodnaviridae*, and 10 from *Caudoviricetes*, representing double-stranded DNA viruses (dsDNA), phage, double-stranded RNA viruses (dsRNA), and single-stranded RNA viruses (ssRNA) (Figure 1). In the virome of *B. incisa*, none of the well-characterized rice viruses were identified. A contig of 6694 nt shared 31.00% nt identity with the L protein of the browner virus, and the predicted protein shared over 65.45% aa identity with the RdRp of Hangzhou nephotettix cincticeps phenuivirus 1, suggesting the presence of one member in *Riboviria*. Four near-complete genome length contigs were chosen for further analysis.

### 3.2. Identification of Two New Members Belonging to the Family Aliusviridae

In the virome of *B. incisa*, we found two contigs shared the highest identity with the protein originating from viruses belonging to the family *Aliusviridae*. A contig of 18,944 nt exhibited the most significant similarities to the RdRp of Taiyuan leafhopper virus (GenBank No. MH708020), while another contig of 15,152 nt displayed the highest sequence similarities to the RdRp of Scaldis River bee virus (GenBank No. KY053857).

**Figure 2:**
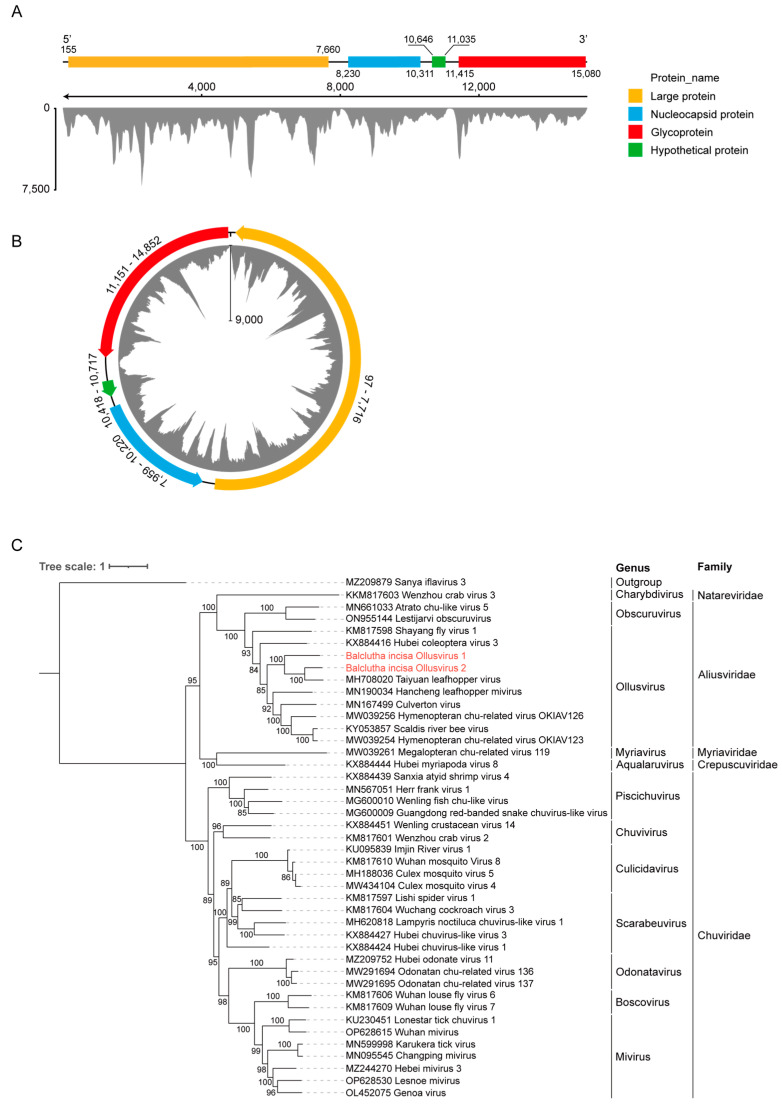
Genome organization and phylogenetic analysis of BiOV1 and BiOV2. Genome length, structure, ORF positions, and sequencing coverage of linear BiOV1 (**A**) and circular BiOV2 (**B**). ORFs are shown as boxes, with colors corresponding to their predicted protein functions. GP, Glycoprotein gene; NP, Nucleoprotein gene; L, Large protein gene; HP, Hypothetical protein gene. (**C**) Phylogenetic analysis based on the conserved sequence of the L protein from members belonging to the family *Aliusviridae* and two novel ollusviruses, with Sanya iflavirus 3 selected as the outgroup. The tree was generated using iqtree2 with the LG + G best-fit model with 1000 bootstraps. Bootstrap values (>60%) are shown at each node of the tree. The bar represents genetic distance. The novel viruses identified in this study are highlighted in red.

Using the 5′ and 3′ RACE-PCR techniques, the full genome sequences of two viruses were successfully obtained, and the two viruses are tentatively named B. incisa ollusvirus 1 (BiOV1) and B. incisa ollusvirus 2 (BiOV2). It is worth noting that the gene sequence recovered from the 5′ RACE of BiOV2 overlaps with its 3′ end. In order to verify the circularity of the genome of BiOV2, we used sequence-specific divergent primers for PCR. The electrophoresis results showed that the divergent primers of BiOV1 did not amplify any bands, while the divergent primers of BiOV2 amplified the corresponding amplicon (Figure S1). The full-length linear genome of BiOV1 (deposited in GenBank under accession number C_AA042969) is 15,125 nt, while the circular genome of BiOV2 (deposited in GenBank under accession number C_AA058669) is 14,853 nt (Figure 2A,B), and both ollusviruses contain four predicted ORFs. In the linear BiOV1 genome, ORF1 (155–7660 nt) encodes a 277.7 kDa L protein with the RdRp domain. ORF2 (8230–10,311 nt) encodes a 77.0 kDa NP, and ORF4 (11,415–15,080 nt) encodes a 135.6 kDa GP. A 14.4 kDa hypothetical protein is encoded by ORF3 (10,646–11,035 nt). In the genome of BiOV2, ORF1 (97–7716 nt) encodes a 292.2 kDa L protein with the RdRp domain, and ORF2 (7959–10,220 nt) encodes an 83.5 kDa NP. ORF3 (10,418–10,717 nt) encodes a 10.6 kDa hypothetical protein, and ORF4 (11,151–14,852 nt) encodes a 135.1 kDa GP. After mapping the sequencing reads to the two virus genomes, a total of 90,092 and 326,131 reads were found in BiOV1 and BiOV2, suggesting a high abundance of these two viruses in *B. insica*. The genome sequence identity between the two ollusviruses is 44.74%, while the aa sequence identity of NP, GP, and L between two ollusviruses is less than 50%. Based on species demarcation criteria from ICTV, which state that members of different Aliusvirid species should have L protein sequences with <90% amino acid identity, our results suggested that BiOV1 and BiOV2 are two different species.

To determine the genetic relationship of two ollusviruses in the order *Jingchuvirales*, a phylogenetic tree was constructed using the amino acid sequences of the L protein from representative members in the order *Jingchuvirales*, with Sanya iflavirus 3 (GenBank No. MZ209879), a virus belonging to the family *Iflaviridae*, as the outgroup. The phylogenetic tree suggested the two newly identified viruses, and the Taiyuan leafhopper virus formed a distinct subgroup in the genus *Ollusvirus* (Figure 2C). The pairwise comparison revealed a 41.25% amino acid sequence identity in the L proteins between BiOV1 and BiOV2. The highest identity between BiOV1 or BiOV2 and other members of the genus *Ollusvirus* is less than 90%. Thus, our results suggest that both ollusviruses are novel members belonging to the genus *Ollusvirus*.

### 3.3. Identification of a Novel Member Belonging to the Family Iflaviridae

**Figure 3:**
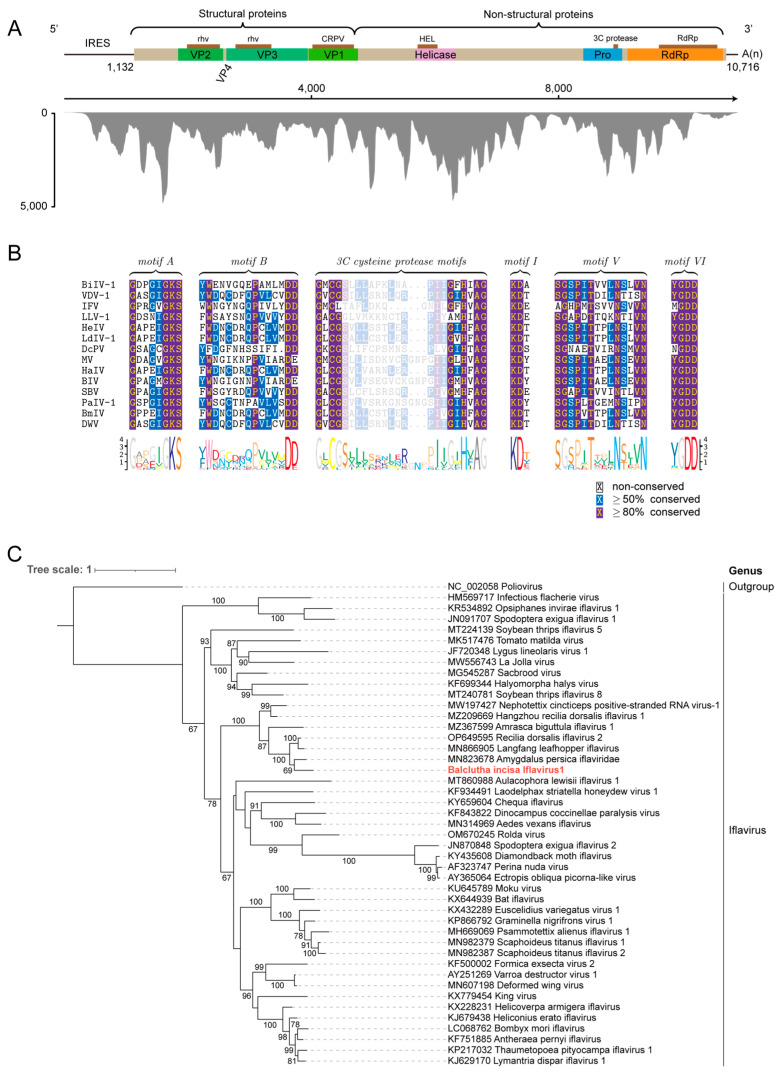
Genome organization and phylogenetic analysis of BiIV1. (**A**) Genome organization of BiIV1. The genome encodes a polyprotein with four structural proteins (VP2-VP4-VP3-VP1) and non-structural proteins containing the HEL (RNA helicase) domain, Pro (3C cysteine protease) domain, and RdRp (RNA-dependent RNA polymerase) domain. The coverage of BiIV1 from the RNA-Seq dataset is plotted below. (**B**) Multiple sequence alignment showing the conserved motifs in representative iflaviruses and BiIV1. (**C**) Maximum-likelihood phylogenetic tree based on the conserved RdRp amino acid sequences of BiIV1 and representative iflaviruses. Poliovirus was used as the outgroup. The phylogenetic tree was generated using iqtree2 with the LG + I + G + F best-fit model and 1000 bootstraps. Bootstrap values (>60%) are shown at each node of the tree. The bar represents the genetic distance. The novel virus identified in this study is highlighted in red.

Another contig of 10,881 nt shares 52.44% identity with its closest analogue, the hypothetical protein of Hubei picorna-like virus 29 (GenBank No. KX883292). The novel virus is named BiIV1 (deposited in GenBank under accession number C_AA042972). Excluding the poly(A) tail, the full-length genome of BiIV1 is 10,903 nt with a GC content of 39%. The genome of BiIV1 contains a single predicted ORF (1132–10,716 nt), encoding a 359.7 kDa polyprotein of 3194 aa. The negative-sense genome contains an 1131 nt 5′ UTR and a 187 nt 3′ UTR. Based on genome structure analysis, BiIV1 possesses the conserved domains typically present in members belonging to the family *Iflaviridae*. The polyprotein contains a typical structure protein arranged in the order VP2-VP4-VP3-VP1 and non-structural proteins in the C-terminal region. The structural proteins located in the N-terminal region include two *rhv* domains (287–462 aa and 549–739 aa) and a CRPV capsid protein-like domain (964–1185 aa), while the non-structural C-terminal region contains a helicase domain (HEL, 1532–1639 aa), a protease domain (Pro), and an RdRp domain (2838–3151 aa) (Figure 3A). Coverage analysis suggested that the sequencing reads cover the entire virus genome, with significantly reduced coverage in the UTRs recovered by RACE. Based on the sequences of iflaviruses from NCBI, conserved motifs were identified [37]. The HEL contains a 3C motif, including motif A with the canonical sequence GxxGxGK(S/T), while motif B ((Y/F/W)2X5QX5(Q/D)D) is not conserved in BiIV1 (Figure 3B). The conserved 3C cysteine protease motifs (GxCG and GxHxxG) between 2592 and 2614 aa suggest the presence of a protease domain. The RdRp of BiIV1 is characterized by three motifs: motif I (KDA), motif V (SGxxxTxxxN(S/T)), and motif VI (YGDD).

To determine the genetic relationship between BiIV1 and other members of the family *Iflaviridae*, a phylogenetic tree was generated based on the conserved RdRp domain region using Poliovirus (GenBank No. NC_002058), a member of the family *Ensavirinae*, as the outgroup. Forty-six members of the family *Iflaviridae* were included, and the maximum likelihood phylogenetic tree indicated that BiIV1, Amygdalus persica iflaviridae (GenBank No. MN823678), and other arthropod iflaviruses clustered together to form a unique branch (Figure 3C). Further sequence identity analysis showed that all capsid proteins of known iflaviruses shared sequence identity lower than 80% with the capsid protein (CP) of BiIV1. According to ICTV, the criteria for species demarcation in the family *Iflaviridae* require that the amino acid sequence identity of the CP be less than 90%. Therefore, we propose that BiIV1 is a novel member belonging to the family *Iflaviridae*, contributing to future investigations of iflaviruses.

### 3.4. Identification of a New Member Belonging to the Family Totiviridae

**Figure 4:**
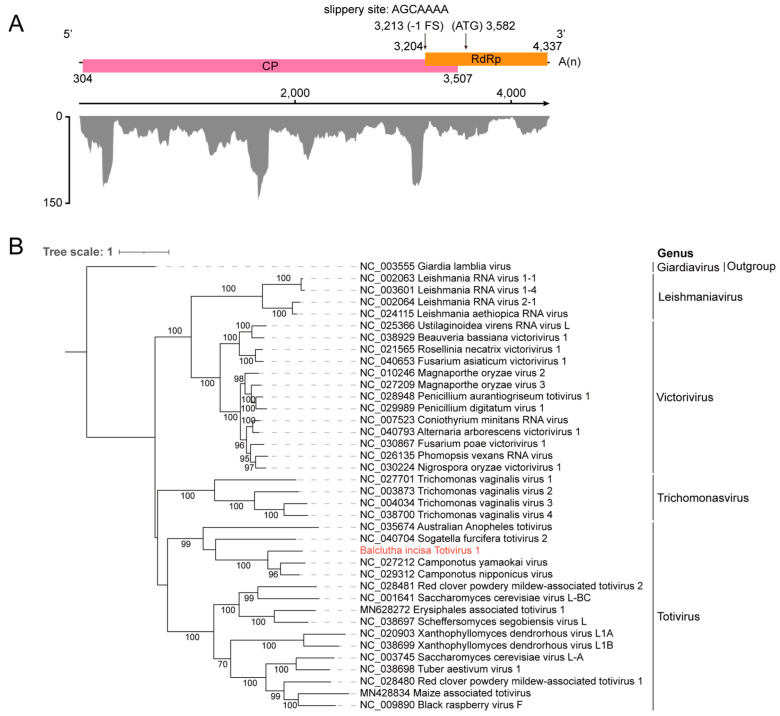
Genome organization and phylogenetic analysis of BiTV1. (**A**) The genome encodes two overlapping proteins: CP and RdRp, and the coverage of raw sequencing reads mapped to the reference genome of BiTV1. (**B**) Phylogenetic analysis based on the aa sequence of the full-length CP from members of different genera in the family *Totiviridae*, with Giardia lamblia virus in the genus *Giardiavirus* serving as the outgroup. The tree was generated using iqtree2 with the Q.pfam + F+R4 best-fit model with 1000 bootstraps. Bootstrap values (>60%) are shown at each node of the tree. The bar represents the genetic distance. The novel virus identified in this study is highlighted in red.

In our results, a contig of 3983 nt shared 37.04% amino acid identity with the CP of Camponotus nipponicus virus (GenBank No. NC_029312). The full genome sequence of BiTV1 (deposited in GenBank under accession number C_AA042970) is 4357 nt, excluding the poly(A) tail. The genome of BiTV1 contains a 303 nt 5′ UTR, two overlapping ORFs, and a 21 nt 3′ UTR (Figure 4). The two overlapping ORFs encode a 1067 aa CP (117.8 kDa) and a 251 aa RdRp (42.3 kDa), respectively. An ORF1-ORF2 fusion protein could be generated via the −1 ribosomal frameshift mechanism (Figure S2) at 3232–3267 nt, similar to other members of the genus *Totivirus* [47]. The abundance and coverage of BiTV1 were evaluated, and only 1485 reads originated from the BiTV1 genome. Based on the amino acid sequences of conserved CP proteins from representative viruses in the families *Totiviridae*, *Chrysoviridae*, *Partitiviridae*, and *Amalgaviridae*, the phylogenetic tree clearly suggested that BiTV1, Camponotus nipponicus virus, Sogatella furcifera totivirus 2 (GenBank No. NC_040704), Camponotus yamaokai virus (GenBank No. NC_027212), and Australian Anopheles totivirus (GenBank No. NC_035674) formed a cluster in the genus *Totivirus* (Figure 4B). Furthermore, the amino acid sequence identity of CP and RdRp between BiTV1 and related viruses is less than 50%. Our results indicated that BiTV1 is a novel member belonging to the family *Totiviridae*.

## 4. Discussion

Rice viruses are among the major threats to global rice production, with leafhoppers playing a pivotal role as vectors in their transmission. In this study, we uncovered the virome of *B. incisa* based on deep sequence analysis and screened and characterized four novel RNA viruses: B. incisa ollusvirus 1 (BiOV1), B. incisa ollusvirus 2 (BiOV2), B. incisa iflavirus (BiIV1), and B. incisa totivirus 1 (BiTV1).

*Jingchuvirales* is a newly established order of negative-sense RNA viruses recognized in 2022 due to their unique genomic structures and distinct evolutionary lineage [33]. Recent research has shown a broad host range for *Jingchuvirales*, especially in insects, fish, and reptiles [33]. While research on *Jingchuvirales* is still emerging, the transmission pathways are not clear. The establishment of this order underscores the importance of genomics in virus taxonomy and highlights the need for further research into their evolutionary history and host interactions, as their roles in host biology and ecosystems have not yet been explored. In this project, we found that the amino acid sequence identity of the L protein between BiOV1 and BiOV2 is less than 90%, suggesting that the two ollusviruses are distinct species. The co-presence of both linear and circular forms of ollusviruses (BiOV1 and BiOV2) in *B. incisa* is interesting, suggesting remarkable genome plasticity or alternative replication strategy within this viral family [48]. The biological function of these two forms of ollusviruses in the host requires further investigation. This finding highlights the need for further research into the mechanisms driving this genomic flexibility and its implications for viral evolution and host interactions.

Members of the family *Iflaviridae* have been reported and characterized as infecting invertebrates, with a particular focus on insects, especially bees, ants, beetles, and butterflies [37]. New members have also been identified in leafhoppers [49,50,51]. Infection in hosts could be asymptomatic or may result in significant pathology. *Iflaviridae* viruses are typically transmitted horizontally between hosts through direct contact, shared food sources, or contaminated environments. Both horizontal and vertical transmission have been observed for the Deformed wing virus (DWV) [52] and Sacbrood virus (SBV) [53]. Furthermore, flowers can act as environmental reservoirs for the transmission of DWV [54]. It is worth noting that tomato matilda virus (GenBank No. MK517476), an iflavirus-like virus, has been reported to infect *Solanum lycopersicum*, *Capsicum annuum*, and *Solanum melongena* [55]. Here, we identified a novel member, BiIV1, in *B. incisa*. The closest relative to BiIV1 in the phylogenetic tree is Amygdalus persica iflaviridae (Gen-Bank No. MN823678), which was found in peach tree samples through sequencing [56]. The transmission pathway and infection mechanisms of BiIV1 remain unclear, and its impact on host species is not yet well understood. However, the genome sequencing and annotation of BiIV1 contribute valuable new insights into the family *Iflaviridae*, expanding our knowledge of this viral group and providing a foundation for future research into its biology and potential effects on insect hosts.

*Totiviridae* is a family of double-stranded RNA (dsRNA) viruses that primarily infect fungi and protozoa, though some members have also been found in arthropods [38,57,58]. The biological significance and impact of *Totivirus* infections in insects remain largely unknown, and the transmission mechanisms within insect hosts are not yet well understood. In this project, we identified a novel virus, BiTV1, a member of the family *Totiviridae*, whose capsid protein (CP) and RNA-dependent RNA polymerase (RdRp) share less than 50% amino acid sequence identity with closely related viruses. This significant divergence highlights the uniqueness of BiTV1 within the *Totivirus* genus and suggests potential differences in its biological properties or host interactions.

In summary, this study unveiled the complete genome sequences of three novel negative-sense RNA viruses and one double-stranded RNA virus within *B. incisa*, marking a significant expansion of our understanding of viral diversity in this insect species. Further studies are essential to elucidate the roles these viruses may play in the ecology of rice pests and their broader impact on agricultural productivity.

## Data Availability

The original contributions presented in this study are included in the article/Supplementary Materials. Further inquiries can be directed to the corresponding authors.

## Supplementary Materials

The following supporting information can be downloaded at https://www.mdpi.com/article/10.3390/insects15100772/s1, Figure S1: Amplification results of the whole genomes of the four novel viruses in this paper; Figure S2: Frame-shifting structure in virus BiTV1; Table S1: List of primers used in this study.
