# Supplementary material for: Identification and Characterization of Four Novel Viruses in Balclutha incisa
Source: Insects. 2024 Oct 6;15(10):772. doi: 10.3390/insects15100772 (PMC11508223; doi:10.3390/insects15100772)
Supplement: Supplementary file 1 [file insects-15-00772-s001.zip › insects-3199497-supplementary.pdf]

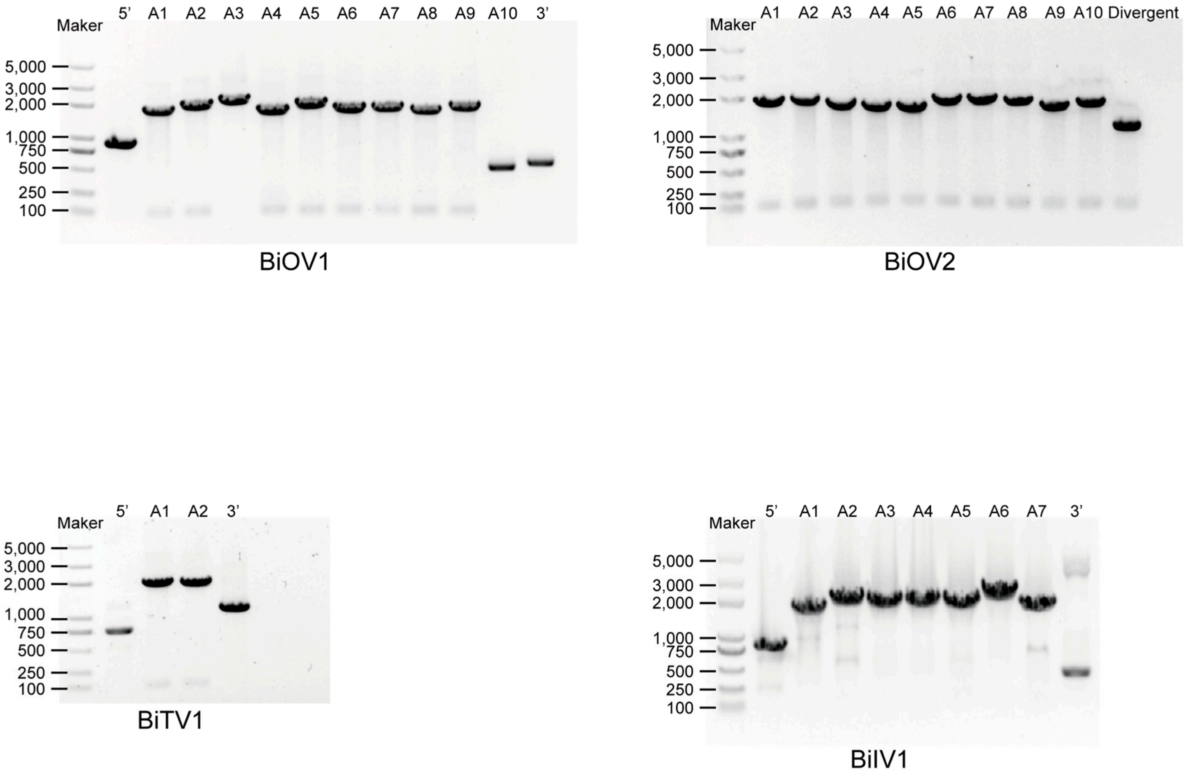

Figure S1. Amplification results of the whole genomes of the four novel viruses in this paper.

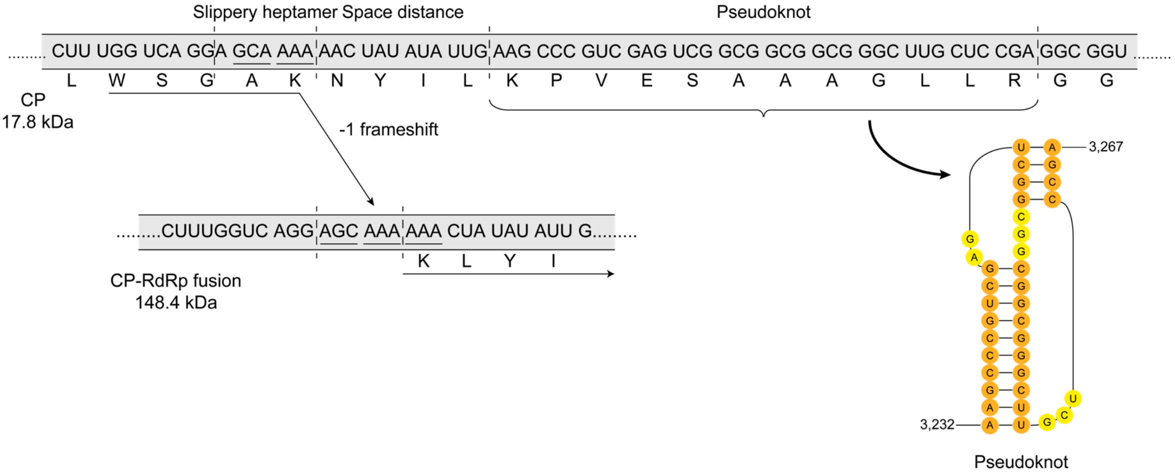

Figure S2. Frame-shifting structure in virus BiTV1.
